# Supplementary material for: Limited role for meteorological factors on the variability in COVID-19 incidence: A retrospective study of 102 Chinese cities
Source: PLoS Negl Trop Dis. 2021 Feb 24;15(2):e0009056. doi: 10.1371/journal.pntd.0009056 (PMC7904227; doi:10.1371/journal.pntd.0009056)
Supplement: S1 Table — (DOCX) [file pntd.0009056.s001.docx]

**S1 Table**. Summary of control measures of Level I response in 27 provinces/municipalities

| **Date of Issue Level I response** | **Province** | **Cities Involved in Analysis** | **Summarized measures of Level I response*** | **Reference** |
| --- | --- | --- | --- | --- |
| 24-Jan | Anhui | Anqing, Bengbu, Bozhou, Fuyang, Hefei, Wuhu | 1. Social distancing 2. Screening and contact tracing 3. Hospital-related measures | [1] |
| 24-Jan | Beijing | Beijing | 1. Social distancing 2. Screening and contact tracing 3. Hospital-related measures | [2] |
| 24-Jan | Chongqing | Chongqing | 1. Social distancing 2. Screening and contact tracing 3. Other public health measures | [3] |
| 24-Jan | Fujian | Fuzhou, Nanping, Ningde, Quanzhou, Xiamen | 1. Social distancing 2. Screening and contact tracing 3. Other public health measures | [4] |
| 25-Jan | Gansu | Lanzhou | 1. Social distancing 2. Other public health measures | [5] |
| 23-Jan | Guangdong | Guangzhou, Jiangmen, Shantou, Shenzhen, Zhanjiang, Zhaoqing | 1. Social distancing 2. Screening and contact tracing 3. Quarantine of risky populations 4. Hospital-related measures 5. Other public health measures | [6] |
| 24-Jan | Guangxi | Beihai, Guilin, Nanning | 1. Social distancing 2. Screening and contact tracing 3. Quarantine of risky populations 4. Hospital-related measures 5. Other public health measures | [7] |
| 24-Jan | Guizhou | Bijie, Guiyang | 1. Screening and contact tracing 2. Quarantine of risky populations 3. Other public health measures | [8] |
| 24-Jan | Hainan | Haikou, Sanya | 1. Social distancing 2. Screening and contact tracing 3. Quarantine of risky populations 4. Other public health measures | [9] |
| 24-Jan | Hebei | Baoding, Cangzhou, Shijiazhuang, Tangshan, Xingtai, Zhangjiakou | 1. Social distancing 2. Screening and contact tracing | [10] |
| 25-Jan | Heilongjiang | Harbin, Jixi, Qiqihar, Shuangyashan, Suihua | 1. Social distancing 2. Screening and contact tracing 3. Quarantine of risky populations 4. Hospital-related measures 5. Other public health measures | [11] |
| 25-Jan | Henan | Anyang, Luoyang, Nanyang, Xinyang, Zhengzhou, Zhoukou, Zhumadian | 1. Social distancing 2. Screening and contact tracing 3. Hospital-related measures 4. Other public health measures | [12] |
| 23-Jan | Hunan | Changde, Changsha, Chenzhou, Hengyang, Huaihua, Shaoyang, Yongzhou, Yueyang | 1. Social distancing 2. Screening and contact tracing 3. Other public health measures | [13] |
| 24-Jan | Jiangsu | Changzhou, Lianyungang, Nanjing, Nantong, Xuzhou, Yancheng | 1. Social distancing 2. Screening and contact tracing 3. Quarantine of risky populations 4. Other public health measures | [14] |
| 24-Jan | Jiangxi | Fuzhou, Ganzhou, Ji'an, Jiujiang, Yichun | 1. Social distancing 2. Quarantine of risky populations 3. Hospital-related measures | [15] |
| 25-Jan | Jilin | Changchun | 1. Screening and contact tracing 2. Other public health measures | [16] |
| 25-Jan | Liaoning | Dalian, Shenyang | 1. Social distancing 2. Screening and contact tracing | [17] |
| 25-Jan | Ningxia | Wuzhong, Yinchuan | 1. Social distancing 2. Screening and contact tracing 3. Hospital-related measures 4. Other public health measures | [18] |
| 25-Jan | Shaanxi | Ankang, Hanzhong, Xi'an | 1. Social distancing 2. Hospital-related measures 3. Other public health measures | [19] |
| 24-Jan | Shandong | Dezhou, Jinan, Jining, Liaocheng, Linyi, Qingdao, Tai'an, Weifang, Weihai, Yantai, Zibo, | 1. Social distancing 2. Screening and contact tracing 3. Other public health measures | [20] |
| 24-Jan | Shanghai | Shanghai | 1. Social distancing 2. Quarantine of risky populations 3. Other public health measures | [21] |
| 25-Jan | Shanxi | Jinzhong, Taiyuan | 1. Social distancing 2. Screening and contact tracing | [22] |
| 24-Jan | Sichuan | Chengdu, Dazhou, Luzhou, Mianyang, Nanchong, Neijiang | 1. Social distancing 2. Screening and contact tracing 3. Hospital-related measures 4. Other public health measures | [23] |
| 24-Jan | Tianjin | Tianjin | 1. Hospital-related measures 2. Other public health measures | [24] |
| 25-Jan | Xinjiang | Urumqi | 1. Social distancing 2. Screening and contact tracing 3. Quarantine of risky populations 4. Other public health measures | [25] |
| 24-Jan | Yunnan | Kunming | 1. Social distancing 2. Screening and contact tracing 3. Quarantine of risky populations 4. Hospital-related measures 5. Other public health measures | [26] |
| 23-Jan | Zhejiang | Hangzhou, Lishui, Ningbo, Quzhou, Shaoxing, Taizhou, Wenzhou | 1. Social distancing 2. Screening and contact tracing 3. Quarantine of risky populations 4. Hospital-related measures 5. Other public health measures | [27] |

*The control measures were grouped into 5 categories. ‘Social distancing’ includes: Cancel of public events, traffic control, suspension of entertainment business, tourism places, factories and schools. ‘Screening and contact tracing’ includes: Body temperature checking at transportation hubs or public venues, registration of risky population, contact tracing of patients, management of close contacts, and enhance screening for risky population. ‘Quarantine of risky populations’ includes: Quarantine of risky travelers, quarantine of close contacts, and quarantine of suspected cases. ‘Hospital-related measures’ includes: Nosocomial transmission control and setup of designated hospitals for suspected COVID-19 patients. ‘Other public health measures’ includes: Public education for disease prevention, clean-up/disinfection of environment, offering free face masks, and forcing face mask wearing in public places.

**Reference**

1. Anhui News. [Internet]. [in Chinese] Anhui launched Level 1 response for major public health emergency: what are the key measures by the government? [cited 2020 July 31]. Available from: https://baijiahao.baidu.com/s?id=1656589486426371595&wfr=spider&for=pc
2. Huanqiu News. [Internet]. [in Chinese] Beijing Government: people from high-risk regions should follow 14-day home quarantine. [cited 2020 July 31]. Available from: https://china.huanqiu.com/article/9CaKrnKp87i
3. Chongqing Daily. [Internet]. [in Chinese] Chongqing Government: full implementation of Level 1 response, control the epidemic. [cited 2020 July 31]. Available from: http://www.cq.xinhuanet.com/2020-01/26/c_1125502425.htm
4. Sohu News. [Internet]. [in Chinese] Most restrict contro! Fujian launched Level 1 response! Fuzhou implemented the below measures. [cited 2020 July 31]. Available from: https://www.sohu.com/a/368860453_120033814
5. Sina Financial News. [Internet]. [in Chinese] Gansu: multiple measures to control the epidemic. [cited 2020 July 31]. Available from: https://baijiahao.baidu.com/s?id=1656701882437827736&wfr=spider&for=pc
6. Dayoo News. [Internet]. [in Chinese] Guangdong launched 16 detailed measures to implement the Level 1 response. [cited 2020 July 31]. https://news.dayoo.com/guangdong/202001/25/139996_53047949.htm
7. Guangxi Daily. [Internet]. [in Chinese] Guangxi implement 18 specific measures to enforce the effort of Level 1 response. [cited 2020 July 31]. Available from: https://baijiahao.baidu.com/s?id=1656790500098354460&wfr=spider&for=pc
8. Guiyang Bendibao. [Internet]. [in Chinese] Guizhou launched Level 1 response for major public health emergency: what shall the residents do? [cited 2020 July 31]. Available from: https://www.sohu.com/a/369067617_822192
9. Tencent News. [Internet]. [in Chinese] Hainan launched Level 1 response for major public health emergency: here are what you want to know. [cited 2020 July 31]. Available from: https://xw.qq.com/cmsid/20200125A068QL00
10. Hebei Daily. [Internet]. [in Chinese] Hebei launched Level 1 response for major public health emergency. [cited 2020 July 31]. Available from: https://www.douban.com/group/topic/163817514/
11. Heilongjiang Province People’s Government. [Internet]. [in Chinese] Heilongjiang launched Level 1 response for major public health emergency. [cited 2020 July 31]. Available from: http://www.hlj.gov.cn/zwfb/system/2020/01/25/010918651.shtml
12. Henan Daily. [Internet]. [in Chinese] Henan launched Level 1 response for major public health emergency. [cited 2020 July 31]. Available from: https://baijiahao.baidu.com/s?id=1656742727739085540&wfr=spider&for=pc
13. Tencent News. [Internet]. [in Chinese] What does it mean to launch Level 1 response in Hunan. [cited 2020 July 31]. Available from: https://new.qq.com/omn/20200125/20200125A0BJ9300?ADTAG=LenovoPC&pgv_ref=LenovoPC&name=LenovoPC
14. China News Net. [Internet]. [in Chinese] Nanjing launched Level 1 response for major public health emergency: specify 7 forced measures. [cited 2020 July 31]. Available from: https://baijiahao.baidu.com/s?id=1656605045715465971&wfr=spider&for=pc
15. Jiangxi Daily. [Internet]. [in Chinese] Jiangxi launched Level 1 response for major public health emergency: what does it mean? [cited 2020 July 31]. Available from: https://baijiahao.baidu.com/s?id=1656605045715465971&wfr=spider&for=pc
16. Sohu News. [Internet]. [in Chinese] Jilin launched Level 1 response for major public health emergency. [cited 2020 July 31]. Available from: http://3g.k.sohu.com/t/n421677545
17. Yingkou Online. [Internet]. [in Chinese] Liaoning launched Level 1 response for major public health emergency: what does it mean. [cited 2020 July 31]. Available from: https://www.sohu.com/a/368894114_120509461
18. Yinchuan Government News. [Internet]. [in Chinese] Ningxia specified 10 measures to control epidemic. [cited 2020 July 31]. Available from: http://www.yinchuan.gov.cn/xwzx/toutiao/202001/t20200125_1932957.html
19. Sina Financial News. [Internet]. [in Chinese] To control the epidemic: Shaanxi launched Level 1 response for major public health emergency. [cited 2020 July 31]. Available from: https://baijiahao.baidu.com/s?id=1656710618995815901&wfr=spider&for=pc
20. Linyi Online. [Internet]. [in Chinese] Shandong’s cumulative confirmed cases reached 21! What shall “I” do according to the Level 1 response/. [cited 2020 July 31]. Available from: https://baijiahao.baidu.com/s?id=1656686620601457782&wfr=spider&for=pc
21. Knews. [Internet]. [in Chinese] Shanghai launched the restricted measures to implement Level 1 response. [cited 2020 July 31]. Available from: https://baijiahao.baidu.com/s?id=1656613055749311826&wfr=spider&for=pc
22. Shanxi Daily. [Internet]. [in Chinese] Shanxi launched Level 1 response! Do the following things! [cited 2020 July 31]. Available from: https://baijiahao.baidu.com/s?id=1656697531077809982&wfr=spider&for=pc
23. People’s Net. [Internet]. [in Chinese] Sichuan launched 8 measures to implement Level 1 response. [cited 2020 July 31]. Available from: http://sc.people.com.cn/n2/2020/0126/c379470-33744625.html
24. Wuqing Life News. [Internet]. [in Chinese] Tianjin launched Level 1 response to face the COVID-19 challenges. [cited 2020 July 31]. Available from: https://m.sohu.com/a/368785569_167770/
25. People’s Net. [Internet]. [in Chinese] Xinjiang: implement Level 1 response, work on epidemic control. [cited 2020 July 31]. Available from: http://xj.people.com.cn/n2/2020/0127/c186332-33746331.html
26. Yunnan Daily. [Internet]. [in Chinese] Yunnan launched Level 1 response for major public health emergency. [cited 2020 July 31]. Available from: http://www.gov.cn/xinwen/2020-01/29/content_5472881.htm
27. Binjiang Publish. [Internet]. [in Chinese] Zhejiang launched Level 1 response and 10 restricted control measures. [cited 2020 July 31]. Available from: <https://baijiahao.baidu.com/s?id=1656595221348480440&wfr=spider&for=pc>
