# Supplementary material for: Limited role for meteorological factors on the variability in COVID-19 incidence: A retrospective study of 102 Chinese cities
Source: PLoS Negl Trop Dis. 2021 Feb 24;15(2):e0009056. doi: 10.1371/journal.pntd.0009056 (PMC7904227; doi:10.1371/journal.pntd.0009056)
Supplement: S2 Table — (DOCX) [file pntd.0009056.s002.docx]

**S2 Table**. Comparison of changes in R-square among different models and relative risks (95% confidence intervals) of the variables when different lags are selected for meteorological factors.

| Variables | M1 (null model) | M3 (lag=3 days) | M5 (full model, lag=3 days) | M3 (lag=7 days) | M5 (full model, lag=7 days) |
| --- | --- | --- | --- | --- | --- |
| City-specific characteristics |  |  |  |  |  |
| Population density (in /100 km^2^) |  | 1.021 (0.996, 1.046) | 1.019 (0.996, 1.043) | 1.019 (0.996, 1.043) | 1.014 (0.992, 1.038) |
| GDP per capita (in 10,000 Chinese Yuan) |  | 1.049 (0.991, 1.110) | 1.021 (0.967, 1.077) | 1.047 (0.991, 1.105) | 1.012 (0.959, 1.067) |
| Proportion of tertiary education (in %) |  | 0.989 (0.948, 1.031) | 1.002 (0.963, 1.043) | 0.994 (0.955, 1.035) | 1.011 (0.972, 1.051) |
| Proportion of elderly population (in %) |  | 0.866 (0.775, 0.967)* | 0.903 (0.812, 1.004) | 0.872 (0.784, 0.969)* | 0.932 (0.840, 1.034) |
| Distances to Wuhan (in 100 km) |  | 0.967 (0.939, 0.996)* | 0.989 (0.961, 1.017) | 0.975 (0.948, 1.003) | 1.007 (0.979, 1.036) |
| Meteorological factors |  |  |  |  |  |
| Temperature (in ^o^C) |  | 0.947 (0.934, 0.960)** | 0.983 (0.968, 0.998)* | 0.957 (0.942, 0.966)** | 1.009 (0.994, 1.025) |
| Relative humidity (in %) |  | 0.998 (0.994, 1.002) | 0.998 (0.993, 1.002) | 1.004 (1.000, 1.008) | 1.000 (0.996, 1.005) |
| Control measure effect |  |  | 0.755 (0.739, 0.771)** |  | 0.753 (0.737, 0.769)** |
| Time trend | 1.009 (1.006, 1.013)** | 1.011 (1.007, 1.014)** | 1.227 (1.206, 1.248)** | 1.009 (1.005, 1.012)** | 1.229 (1.208, 1.250)** |
| χ^2^/*df* | 0.33 | 0.32 | 0.11 | 0.32 | 0.11 |
| *R^2^_fixed_* | 0.98% | 10.4% | 45.6% | 9.15% | 44.7% |
| *R^2^_random_* | 22.7% | 24.4% | 13.7% | 23.0% | 13.4% |
| *∆R^2^_fixed_* | - | 9.41% | 44.7% | 8.17% | 43.7% |

Note: M1, Model with time only; M3 (lag=3 days), Model with city-specific characteristics, meteorological factors at a 3-day lag, and time; M5 (full model, lag=3 days), Model with city-specific characteristics, meteorological factors at a 3-day lag, control measure variable, and time; M3 (lag=7 days), Model with city-specific characteristics, meteorological factors at a 7-day lag, and time; M5 (full model, lag=7 days), Model with city-specific characteristics, meteorological factors at a 7-day lag, control measure variable, and time

RR, Relative risk in incidence rate of COVID-19 for each unit change of variable; χ^2^/*df*, chi-square statistics divided by the degree of freedom; *R^2^_fixed_*, Proportion of variance in the incidence rate (per million population) explained by the fixed effect terms; *R^2^_random_*, Proportion of variance explained by the random effect term of cities’ heterogeneity. *∆R^2^_fixed_*, *R^2^_fixed_* of each model minus *R^2^_fixed_* of M1.

**p*<0.05; ***p*<0.001
