# Supplementary material for: Limited role for meteorological factors on the variability in COVID-19 incidence: A retrospective study of 102 Chinese cities
Source: PLoS Negl Trop Dis. 2021 Feb 24;15(2):e0009056. doi: 10.1371/journal.pntd.0009056 (PMC7904227; doi:10.1371/journal.pntd.0009056)
Supplement: S3 Table — (DOCX) [file pntd.0009056.s003.docx]

**S3 Table**. Changes of R-square and relative risks (95% confidence intervals) of the variables when interaction terms were included

| Variables | Relative risks (95% confidence intervals) |
| --- | --- |
| City-specific characteristics |  |
| Population density (in /100 km^2^) | 1.019 (0.995-1.043) |
| GDP per capita (in 10,000 Chinese Yuan) | 1.021 (0.967-1.078) |
| Proportion of tertiary education (in %) | 1.000 (0.960-1.041) |
| Proportion of elderly population (in %) | 0.905 (0.813-1.007) |
| Distances to Wuhan (in 100 km) | 0.985 (0.957-1.014) |
| Meteorological factors |  |
| Temperature (in ^o^C) | 0.991 (0.975-1.007) |
| Relative humidity (in %) | 0.990 (0.984-0.995)* |
| Control measure effect | 0.741 (0.713-0.769)** |
| Temperature*Policy effect | 0.999 (0.998-1.000) |
| Relative humidity* Policy effect | 1.000 (1.000-1.001) |
| Time trend | 1.224 (1.203-1.246)** |
| χ^2^/*df* | 0.11 |
| *R^2^_fixed_* | 46.2% |
| *R^2^_random_* | 13.8% |
| *∆R^2^_fixed_* | 45.2% |

RR: Relative risk in incidence rate of COVID-19 for each unit change of variable; χ^2^/*df*: chi-square statistics divided by the degree of freedom; *R^2^_fixed_*: Proportion of variance in the incidence rate (per million population) explained by the fixed effect terms; *R^2^_random_*: Proportion of variance explained the random effect term of cities’ heterogeneity. *∆R^2^_fixed_*: *R^2^_fixed_* of each model minus *R^2^_fixed_* of M1.

**p*-value<0.05; ** *p*-value<0.001
