# Supplementary material for: Limited role for meteorological factors on the variability in COVID-19 incidence: A retrospective study of 102 Chinese cities
Source: PLoS Negl Trop Dis. 2021 Feb 24;15(2):e0009056. doi: 10.1371/journal.pntd.0009056 (PMC7904227; doi:10.1371/journal.pntd.0009056)
Supplement: S4 Table — (DOCX) [file pntd.0009056.s004.docx]

**S4 Table**. Changes of R-square and relative risks (95% confidence intervals) of the variables when day-of-week was included

| Variables | Relative risks (95% confidence intervals) |
| --- | --- |
| City-specific characteristics |  |
| Population density (in /100 km^2^) | 1.019 (0.995-1.047) |
| GDP per capita (in 10,000 Chinese Yuan) | 1.021 (0.966-1.078) |
| Proportion of tertiary education (in %) | 1.000 (0.961-1.042) |
| Proportion of elderly population (in %) | 0.907 (0.815-1.010) |
| Distances to Wuhan (in 100 km) | 0.987 (0.958-1.016) |
| Meteorological factors |  |
| Temperature (in ^o^C) | 0.985 (0.969-0.999)* |
| Relative humidity (in %) | 0.993 (0.988-0.997)* |
| Control measure effect | 0.754 (0.738-0.771)** |
| Day of Week | 0.989 (0.964-1.015) |
| Time trend | 1.227 (1.206-1.248)** |
| χ^2^/*df* | 0.11 |
| *R^2^_fixed_* | 45.8% |
| *R^2^_random_* | 13.9% |
| *∆R^2^_fixed_* | 44.8% |

RR: Relative risk in incidence rate of COVID-19 for each unit change of variable; χ^2^/*df*: chi-square statistics divided by the degree of freedom; *R^2^_fixed_*: Proportion of variance in the incidence rate (per million population) explained by the fixed effect terms; *R^2^_random_*: Proportion of variance explained the random effect term of cities’ heterogeneity. *∆R^2^_fixed_*: *R^2^_fixed_* of each model minus *R^2^_fixed_* of M1.

**p*-value<0.05; ** *p*-value<0.001
