# Supplementary material for: Limited role for meteorological factors on the variability in COVID-19 incidence: A retrospective study of 102 Chinese cities
Source: PLoS Negl Trop Dis. 2021 Feb 24;15(2):e0009056. doi: 10.1371/journal.pntd.0009056 (PMC7904227; doi:10.1371/journal.pntd.0009056)
Supplement: S1 Text — (DOCX) [file pntd.0009056.s005.docx]

**S1 Text.** Generalized linear mixed effect models (GLMMs) for different types of control measures

Using a similar modelling framework in the main text, we denote *y_ij_* as the daily incidence rate on day *j* in city *i* (i.e. $n^{onset}_{ij}$ /population size of city *i*), follows a Poisson distribution with mean *λ_ij_*. The full model form is as follow:

$$\ln(\lambda_{ij})=\beta_{0}+\sum_{p} \beta_{p}x_{p_{i}}+\sum_{q} \beta_{q}x_{q_{ij}}+\sum_{d} \beta_{d}x_{d_{ij}}+\beta_{t}time_{i}+\alpha_{i}$$

where *β_0_* is the grand intercept, $x_{p_{i}}$is the *p*-th city-specific characteristic variable of city *i* with regression coefficient *β_p_*, $x_{q_{ij}}$is the *q*-th time-varying meteorological variable of city *i* on day *j* with regression coefficient *β_q_*, $x_{d_{ij}}$is the variable with regression coefficient *β_d_* which captures the incremental effect of city-specific control measure type *d* (*d*=social distancing, screening and contact tracing, quarantine of risky populations, hospital-related measures, or other public health measures) implemented on day *k.* $x_{d_{ij}}$is defined as below:

$$x_{d_{ij}}=\left\{\begin{matrix} 0\text{ where }j\leq\text{date of implementing control measure type }d\text{ in city }i \\ j-k\text{ where }j>\text{date of implementing control measure type }d\text{ in city }i\text{ } \end{matrix}\right.$$

Variable *time_i_* is a time trend variable which is the number of days since the date of the first case with illness in city *i* with regression coefficient *β_t_* in the model. In the GLMM, the city-specific random effect is modelled as *α_i_* which followed a normal distribution with mean 0 and variance *σ_α_^2^*. The use of the random effect is to capture the city-specific heterogeneity that cannot be accounted for by our data. To account for over-dispersion of the outcome variable, *y_ij_* was assumed to follow a negative binomial distribution when the standard Pearson chi-squared statistic divided by its residual degree of freedom (χ^2^/*df*) was greater than two.
