# Supplementary material for: Limited role for meteorological factors on the variability in COVID-19 incidence: A retrospective study of 102 Chinese cities
Source: PLoS Negl Trop Dis. 2021 Feb 24;15(2):e0009056. doi: 10.1371/journal.pntd.0009056 (PMC7904227; doi:10.1371/journal.pntd.0009056)
Supplement: Attachment — Submitted filename: Manuscript.docx [file pntd.0009056.s006.docx]

**Limited role for meteorological factors on the variability in COVID-19 incidence: A retrospective study of 102 Chinese cities**

Ka Chun Chong^1,2,3^, Jinjun Ran^4^, Steven Yuk Fai Lau^1^, William Bernard Goggin^1^, Shi Zhao^1,2^, Pin Wang^5^, Linwei Tian^4^, Maggie Haitian Wang^1,2^, Kirran N. Mohammad^1^, Lai Wei^1^, Xi Xiong^1^, Hengyan Liu^1^, Yawen Wang^1^, Paul Kay Sheung Chan^6^, Jingxuan Wang^1^*

^1^Jockey Club School of Public Health and Primary Care, The Chinese University of Hong Kong, Hong Kong Special Administrative Region, China

^2^Clinical Trials and Biostatistics Laboratory, Shenzhen Research Institute, The Chinese University of Hong Kong, China

^3^Centre for Health System and Policy Research, The Chinese University of Hong Kong, Hong Kong Special Administrative Region, China

^4^School of Public Health, Li Ka Shing Faculty of Medicine, University of Hong Kong, Hong Kong Special Administrative Region, China

^5^Yale School of Public Health, Yale University, United States

^6^Department of Microbiology, Faculty of Medicine, Chinese University of Hong Kong, Hong Kong Special Administrative Region, China

* **Corresponding author:**

**Email:** jxwang@link.cuhk.edu.hk

**Short title:** Limited role for meteorological factors in COVID-19

**Abstract**

While many studies have focused on identifying the association between meteorological factors and the activity of COVID-19, we argue that the contribution of meteorological factors to a reduction of the risk of COVID-19 was minimal when the effects of control measures were taken into account. In this study, we assessed how much variability in COVID-19 activity is attributable to city-level socio-demographic characteristics, meteorological factors, and the control measures imposed. We obtained the daily incidence of COVID-19, city-level characteristics, and meteorological data from a total of 102 cities situated in 27 provinces/municipalities outside Hubei province in China from 1 January 2020 to 8 March 2020, which largely covers almost the first wave of the epidemic. Generalized linear mixed effect models were employed to examine the variance in the incidence of COVID-19 explained by different combinations of variables. According to the results, including the control measure effects in a model substantially raised the explained variance to 39.5%, which increased by around one-third compared to the null model that did not include any covariates. On top of that, including temperature and relative humidity in the model could only result in < 1% increase in the explained variance even though temperature showed a statistically significant association with the incidence rate of COVID-19. In conclusion, we showed that very limited variability of the COVID-19 incidence was attributable to meteorological factors. Instead, the control measures could explain a larger proportion of variance.

**Author summary**

COVID-19 has a great impact worldwide, especially in some rural settings where healthcare resources are not sufficient. While control measures in these area may be limited, scholars have been discussing the potential effects of meteorological factors on mitigating COVID-19 transmission. Unfortunately, the majority of literatures only looked at the association between COVID-19 and environmental factors in which their findings could mislead readers that certain environmental conditions could be ‘protective’. In this study, we argue that the impact of the meteorological factors was very limited by using the incidence data from 102 Chinese cities in the first epidemic period when control measures have been taken into account. As what we expected, once the control measures have been incorporated in the modelling analysis, the meteorological factors could only explain < 1% increase in variability of COVID-19 while control measure explained the variance for more than one-third in total. Because of it, we suggest stringent control measures are necessary to control COVID-19 regardless the meteorological conditions of an area. Given that no vaccine is available to date, our investigation provides an additional evidence, as advocated by World Meteorological Organization rather than relying on changes in the natural environment for mitigation, active non-pharmaceutical interventions are necessary to curb the COVID-19 pandemic.

**Introduction**

Pneumonia cases associated with a novel coronavirus were first recognised at the end of December 2019 in Wuhan City, Hubei Province of China. The 2019 coronavirus disease (COVID-19) soon spread to all 34 provinces of China by the end of January. In response to this epidemic, a lockdown was imposed in Wuhan city starting from 23 January 2020, followed by travel restrictions in Hubei. By 29 January 2020, a total of 7,711 confirmed cases and 170 deaths were diagnosed in China, and 31 provinces/municipalities had launched the highest level (level I) of response for major public health emergencies [1] which aimed at preventing and controlling the emergency, to guide and standardize emergency-handling strategies and to minimize the harm caused by such emergency in a prompt and effective manner [2]. The control measure strategies covered nine main medical, social and political aspects: direct leading from the State Council, definition of risk areas, screening of the floating population, traffic control, social distancing, resource mobilization, information release, public education and maintaining social stability. Despite the restrictive political and societal interventions, the disease soon becomes a global public health threat. As of 11 March 2020, the World Health Organization (WHO) reported 118,319 confirmed cases and 4,292 deaths in over 100 countries/regions [3] and announced COVID-19 as a global pandemic on the same day [4].

Scholars have been discussing the potential effects of meteorological factors on COVID-19 transmission. Previous influenza studies found that in cold and dry weather, respiratory droplets remain airborne longer, the virus is more stable and hosts tend to have weakened immunity, which therefore facilitate virus transmission [5]. Existing laboratory data also suggested that severe acute respiratory syndrome coronavirus 2 (SARS-CoV-2) was more stable at a low temperature [6]. Yet, among the population-based studies, the meteorological effects were inconsistent [7-12] and none have assessed the extent to which the effect contributed to the variability of COVID-19 incidence. It was argued that the inconsistent findings may be due to the decreasing impacts of meteorological conditions after the imposition of political and societal measures for epidemic control [12].

While many studies have focused on identifying the association between meteorological factors and the activity of COVID-19, we hypothesize that the impact of the meteorological factors on risk of COVID-19 was minimal when the effects of control measures were taken into account. In this study, we aim to use data from the first wave of epidemic in China (outside Hubei) to assess how much variability in COVID-19 activity is attributable to city-level socio-demographic characteristics, meteorological factors, and control measures of level I responses. We argue stringent control measures are necessary to control COVID-19 regardless the meteorological conditions of an area.

**Method**

**Settings and primary data screening**

We obtained data from a total of 102 cities situated in 27 provinces/municipalities outside Hubei province in China from 1 January 2020 to 8 March 2020, which largely covers almost the first wave of the epidemic. These cities were selected given that at least 20 cases of COVID-19 were confirmed during the study period. Hubei province as an epidemic centre exhibited completely different epidemic growth compared to other locations and hence was excluded from the analysis. Fig 1 shows the spatial distribution of the 102 Chinese cities selected in this study.

**Fig 1. The 102 Chinese cities selected in the study.**

**Level I responses in different Chinese provinces and municipalities**

By 25 January 2020, all 27 provinces in our study had initiated the level I response to major public health emergencies. The schedule of level I responses and corresponding control measures in each province/municipality were summarized in S1 Table. As a follow-up to the provincial response, many cities launched more specific and multi-dimensional measures. For example, closure of museums, tourist area and religious institutes in Hangzhou, Zhejiang Province, forced masks-wearing and body temperature-checking in public spaces in Shenzhen, Guangdong Province, as well as cancelling mass gathering activities and screening for individuals with travel history from Hubei in Quanzhou, Fujian Province.

**COVID-19 surveillance data**

Daily number of confirmed cases of COVID-19 in different Chinese cities from 1 January 2020 to 8 March 2020 were obtained from the webpage of the National Health Commissions of the People's Republic of China [13]. We employed daily incidence, which is defined as the number of cases with illness onset on that day divided by the population size (per million population) in a city, to describe the activity of COVID-19.

To adjust for the delay between date of illness onset and date of confirmation of COVID-19 diagnosis, we rebuilt the epidemic curves using the following recurrence equation:

(1)

where$n^{onset}_{i}(j)$ and $n^{confirm}_{i}(j)$are the number of cases with onset day *j* and the number of cases confirmed on day *j* in city *i* respectively, and$\varphi(u)$ is the discretized probability density function of delay duration *U* that was assumed to follow a gamma distribution with mean of 8.8 days and standard deviation of 4.6 days between 1 and 27 January 2020 and mean of 5.3 days and standard deviation of 3.0 days from 28 January 2020 onwards [14].

**Meteorological data and other covariates**

Daily meteorological data including average ambient temperature and relative humidity in each of the cities were collected from the National Climate Data Center [15] and were averaged over all weather stations in a city. As absolute humidity has been demonstrated to have a stronger association with respiratory diseases [16-19], we employed vapour pressure determined by Clausius–Clapeyron equation as a proxy for absolute humidity in our analysis [20-22].

In order to account for the variability between cities in our analysis, we also collected city-specific characteristics including population size, population density, sociodemographic status (i.e. gross domestic product (GDP) per capita, proportion of individuals having tertiary education or above, and proportion of elderly population (i.e. aged >64 years) [23], and geographic distance to Wuhan, which served as a proxy for potential accessibility to the epidemic centre.

**Statistical analysis**

To account for between-city variation, we employed generalized linear mixed effect models (GLMMs) to examine the variability in the incidence of COVID-19 explained by different combinations of variables. Suppose *y_ij_*, the daily incidence rate on day *j* in city *i* (i.e. $n^{onset}_{ij}$ /population size of city *i*), follows a Poisson distribution with mean *λ_ij_*, the full model form is as follow:

(2)

where *β_0_* is the grand intercept, $x_{p_{i}}$is the *p*-th city-specific characteristic variable of city *i* with regression coefficient *β_p_*, $x_{q_{ij}}$is the *q*-th time-varying meteorological variable of city *i* on day *j* with regression coefficient *β_q_*, $x_{m_{ij}}$is the variable with regression coefficient *β_m_* which captures the incremental effect of control measures of level I responses implemented on day *k* as defined below:

.

(3)

To account for the time trend, variable *time_i_*, which is the number of days since the study start date with regression coefficient *β_t_*, was included in the model. In the GLMM, the city-specific random effect is modelled as *α_i_* which followed a normal distribution with mean 0 and variance *σ_α_^2^*. To account for over-dispersion of the outcome variable, *y_ij_* was assumed to follow a negative binomial distribution when the standard Pearson chi-squared statistic divided by its residual degree of freedom (χ^2^/*df*) was greater than two.

We compared five regression models: (i) model with time trend only (M1, base model), (ii) model with city-specific characteristics and time trend (M2), (iii) model with city-specific characteristics, meteorological factors, and time trend (M3), (iv) model with city-specific characteristics, control measures variable, and time trend (M4), and (v) model with city-specific characteristics, meteorological factors, control measures variable, and time trend (M5, full model) using R-squares (*R^2^*) proposed by Nakagawa and colleagues [24], so as to determine which variable combination best explains the variance of the activity of SARS-CoV-2. We used *R^2^_fixed_* to depict the proportion of variance explained by the fixed effects and *R^2^_random_* to depict the proportion of variance explained by the random effects of cities’ heterogeneity. *∆R^2^_fixed_* was used to determine the proportion of variance explained by the additional fixed effect terms in each of the M2 to M5 compared with M1. To avoid the problem of collinearity, the impact of vapour pressure was studied in another set of models by replacing temperature and relative humidity with vapour pressure. Relative risks (RR) with corresponding 95% confidence intervals (CIs) and *p*-values (*p*) were employed to quantify the effects of the variables on risk of COVID-19.

In the sensitivity analysis, we tested whether adding an interaction term between meteorological factors and control measures in the model would enhance the explained variance. We also compared the main results from models using different lags for meteorological factors (i.e. 3 and 7 lag days) to assess the robustness of our findings. All analyses were carried out using software SAS version 9.4.

**Ethics statement**

This is a retrospective modelling analysis using publicly available data in a daily aggregated scale. Hence, there are no specific ethical considerations.

**Results**

Table 1 shows the descriptive statistics of the city-specific characteristics of the selected 102 cities. The population size ranged from around 600 thousand (Sanya, Hainan province) to 34 million (Chongqing municipality), whereas the population density ranged from 66/km^2^ (Wuzhong, Ningxia province) to 6,523/km^2^ (Shenzhen, Guangdong province). The GDP per capita and distance to Wuhan ranged from 22 thousand to 190 thousand Chinese yuan and 210 km to 3,270 km respectively. Beijing municipality had the largest proportion of residents with tertiary education (42.3%), while Chongqing had the largest proportion of population aged >64 years (12.9%).

**Table 1**. Descriptive statistics of city-specific characteristics in 102 cities.

|  | Minimum | 25^th^ percentile | Median | 75^th^ percentile | Maximum |
| --- | --- | --- | --- | --- | --- |
| Population (in 10,000) | 60 | 413 | 605 | 825 | 3,397 |
| Population density (in /km^2^) | 66 | 254 | 514 | 706 | 6,523 |
| GDP per capita (in 10,000 Chinese Yuan) | 2.2 | 3.9 | 6.3 | 8.8 | 19.0 |
| Proportion of individuals having tertiary education (%) | 8.5 | 10.9 | 12.0 | 13.7 | 42.3 |
| Proportion of elderly population (%) | 7.4 | 9.5 | 10.8 | 11.7 | 12.9 |
| Distances to Wuhan (in 100 km) | 2.1 | 6.7 | 9.3 | 12.5 | 32.7 |

Of the included cities, the overall median temperature was 6.89^o^C (range: -15.03^o^C to 22.64^o^C) and the median temperature increased from 4.76^o^C (range: -18.45^o^C to 22.24^o^C) in January 2020 to 9.97^o^C (range: -7.38^o^C to 24.26^o^C) in March 2020 (Fig 2A). The overall median relative humidity was 74.42% (Range: 44.87% to 89.73%) and the median relative humidity decreased slightly from 76.29% (range: 51.62% to 90.85%) in January 2020 to 73.81% (range: 30.31% to 97.85%) in March 2020 (Fig 2B).

Before 22 January 2020, most of the cities had a daily incidence rate below 2 per million population (Fig 2C). Before further upsurge of epidemic outbreaks, the Chinese provincial governments have declared level I responses during 23 to 25 January 2020. After that, Shenzhen in Guangdong province experienced the peak daily incidence of 6.9 per million inhabitants on 28 January 2020 among all cities outside Hubei province. A downward trend was observed in the epidemic curve about a week after the implementation of control measures of level I responses.

**Figure 2. Temporal distribution of (A) ambient temperature (^o^C), (B) relative humidity (%), (C) vapour pressure (hPa), and (D) incidence of COVID-19 infections (per million population).** The level I responses were implemented in different provinces between 23 and 25 January 2020.

Table 2 shows the model comparison results. Compared with M1, which solely included the time trend, including city-specific characteristics in the model (M2) could only explain an additional 3.54% of the variance in the incidence rate. Further inclusion of temperature and relative humidity as time-varying fixed effects in the model (M3) boosted the explained variance to 20.8%. However, having the control measure effects included in the GLMM (M4) substantially raised the explained variance to 39.5%, which increased by around one-third compared to the null model. On top of this effect, including the meteorological effects in the model (M5) only resulted in < 1% increase in the explained variance even though temperature showed a statistically significant association with the incidence rate of COVID-19 (RR=0.958, 95% CI: 0.944-0.973, *p*<0.001). Distance to Wuhan (RR=0.965, 95% CI: 0.937-0.994, *p*=0.03) and proportion of elderly population (RR=0.875, 95% CI: 0.784-0.976, p=0.03) were marginally associated with the COVID-19 incidence.

**Table 2**. Comparison of changes in R-square among different models and relative risks (95% confidence intervals) of the variables.

| Variables | M1 (null model) | M2 | M3 | M4 | M5 (full model) |
| --- | --- | --- | --- | --- | --- |
| City-specific characteristics |  |  |  |  |  |
| Population density (in /100 km^2^) |  | 1.015 (0.994, 1.037) | 1.029 (1.000, 1.057)* | 1.017 (0.995, 1.039) | 1.024 (0.999, 1.048) |
| GDP per capita (in 10,000 Chinese Yuan) |  | 1.045 (0.994, 1.099) | 1.071 (1.005, 1.141)* | 1.050 (0.998, 1.105) | 1.063 (1.005, 1.124) |
| Proportion of tertiary education (in %) |  | 1.012 (0.976, 1.050) | 0.986 (0.940, 1.033) | 1.011 (0.974, 1.049) | 0.999 (0.958, 1.041) |
| Proportion of elderly population (in %) |  | 0.918 (0.833, 1.011) | 0.837 (0.740, 0.948)* | 0.919 (0.834, 1.014) | 0.875 (0.784, 0.976)* |
| Distances to Wuhan (in 100 km) |  | 1.00 (0.975, 1.025) | 0.946 (0.915, 0.978)* | 0.993 (0.968, 1.019) | 0.965 (0.937, 0.994)* |
| Meteorological factors |  |  |  |  |  |
| Temperature (in ^o^C) |  |  | 0.925 (0.912, 0.939)** |  | 0.958 (0.944, 0.973)** |
| Relative humidity (in %) |  |  | 0.991 (0.987, 0.995)** |  | 0.998 (0.994, 1.002) |
| Control measure effect |  |  |  | 0.805 (0.791, 0.819)** | 0.808 (0.794, 0.822)** |
| Time trend | 0.993 (0.992, 0.994)** | 0.993 (0.992, 0.994)** | 0.995 (0.994, 0.996)** | 1.219 (1.199, 1.240)** | 1.217 (1.196, 1.237)** |
| χ^2^/*df* | 0.36 | 0.36 | 0.32 | 0.19 | 0.18 |
| *R^2^_fixed_* | 8.34% | 11.9% | 20.8% | 39.5% | 40.3% |
| *R^2^_random_* | 20.0% | 16.8% | 23.2% | 12.0% | 14.1% |
| *∆R^2^_fixed_* | - | 3.54% | 12.4% | 31.1% | 31.9% |

Note: M1, Model with time only; M2, Model with city-specific characteristics and time; M3, Model with city-specific characteristics, meteorological factors, and time; M4, Model with city-specific characteristics, control measure variable, and time; M5, Model with city-specific characteristics, meteorological factors, control measure variable, and time (full model). RR, Relative risk in incidence rate of COVID-19 for each unit change of variable; χ^2^/*df*, chi-square statistics divided by the degree of freedom; *R^2^_fixed_*, Proportion of variance in the incidence rate (per million population) explained by the fixed effect terms; *R^2^_random_*, Proportion of variance explained by the random effect term of cities’ heterogeneity. *∆R^2^_fixed_*, *R^2^_fixed_* of each model minus *R^2^_fixed_* of M1.

When temperature and relative humidity in the models were replaced with vapour pressure, the increases in explained variance were similar (Table 3). Nevertheless, a decrease in vapour pressure was statistically significantly associated with an increased risk of the COVID-19 incidence (RR=0.958, 95% CI: 0.940-0.976, *p*<0.001).

**Table 3**. Comparison of changes in R-square among different models and relative risks (95% confidence intervals) of the variables when vapour pressure was used to replace temperature and relative humidity.

| Variables | M1 (null model) | M3 | M5 (full model) |
| --- | --- | --- | --- |
| City-specific characteristics |  |  |  |
| Population density (in /100 km^2^) |  | 1.026 (1.000, 1.053) | 1.020 (0.997, 1.044) |
| GDP per capita (in 10,000 Chinese Yuan) |  | 1.058 (0.996, 1.125) | 1.055 (1.000, 1.113) |
| Proportion of tertiary education (in %) |  | 0.992 (0.948, 1.037) | 1.003 (0.964, 1.044) |
| Proportion of elderly population (in %) |  | 0.845 (0.751, 0.951)* | 0.894 (0.805, 0.992)* |
| Distances to Wuhan (in 100 km) |  | 0.981 (0.951, 1.011) | 0.986 (0.960, 1.013) |
| Meteorological factors |  |  |  |
| Absolute humidity (in hPa) |  | 0.890 (0.873, 0.908)** | 0.958 (0.940, 0.976) ** |
| Control measure effect |  |  | 0.809 (0.795, 0.823)** |
| Time trend | 0.993 (0.992, 0.994)** | 0.994 (0.993, 0.995)** | 1.214 (1.194, 1.235)** |
| χ^2^/*df* | 0.36 | 0.32 | 0.18 |
| *R^2^_fixed_* | 8.34% | 19.0% | 39.5% |
| *R^2^_random_* | 20.0% | 22.2% | 13.4% |
| *∆R^2^_fixed_* | - | 10.6% | 31.1% |

Note: M1, Model with time only; M3, Model with city-specific characteristics, vapour pressure, and time M5, Model with city-specific characteristics, vapour pressure, control measure variable, and time (full model). RR, Relative risk in incidence rate of COVID-19 for each unit change of variable; χ^2^/*df*, chi-square statistics divided by the degree of freedom; *R^2^_fixed_*, Proportion of variance in the incidence rate (per million population) explained by the fixed effect terms; *R^2^_random_*, Proportion of variance explained by the random effect term of cities’ heterogeneity. *∆R^2^_fixed_*, *R^2^_fixed_* of each model minus *R^2^_fixed_* of M1.

As shown in the sensitivity analysis, our results were robust to the delayed effects of meteorological factors (Table 4). When the lags of temperature and relative humidity were increased in the models, a slight improvement in the explained variance was observed (i.e. *R^2^_fixed_*=22.1% in M3, lag=3 days and *R^2^_fixed_*=24.8% in M3, lag=7 days). However, compared with *R^2^_fixed_* of M4, *R^2^_fixed_* of the full model that accounted for lag effects did not rise remarkably and was kept at around 40%. Adding interaction term in the model did not enhance the proportion of variance explained (S2 Table).

**Table 4**. Comparison of changes in R-square among different models and relative risks (95% confidence intervals) of the variables when different lags are selected for meteorological factors.

| Variables | M1 (null model) | M3 (lag=3 days) | M5 (full model, lag=3 days) | M3 (lag=7 days) | M5 (full model, lag=7 days) |
| --- | --- | --- | --- | --- | --- |
| City-specific characteristics |  |  |  |  |  |
| Population density (in /100 km^2^) |  | 1.030 (1.002, 1.059)* | 1.024 (1.000, 1.050) | 1.030 (1.002, 1.060)* | 1.022 (1.000, 1.046) |
| GDP per capita (in 10,000 Chinese Yuan) |  | 1.073 (1.006, 1.144)* | 1.066 (1.007, 1.128)* | 1.074 (1.007, 1.146)* | 1.061 (1.005, 1.119)* |
| Proportion of tertiary education (in %) |  | 0.988 (0.942, 1.036) | 0.997 (0.956, 1.040) | 0.990 (0.944, 1.039) | 1.003 (0.964, 1.043) |
| Proportion of elderly population (in %) |  | 0.823 (0.726, 0.932)* | 0.872 (0.780, 0.974)* | 0.816 (0.719, 0.925)* | 0.882 (0.794, 0.980)* |
| Distances to Wuhan (in 100 km) |  | 0.944 (0.913, 0.976)* | 0.963 (0.935, 0.993)* | 0.942 (0.911, 0.974)* | 0.975 (0.948, 1.003) |
| Meteorological factors |  |  |  |  |  |
| Temperature (in ^o^C) |  | 0.916 (0.903, 0.929)** | 0.956 (0.941, 0.970)** | 0.909 (0.897, 0.921)** | 0.969 (0.955, 0.984)** |
| Relative humidity (in %) |  | 0.998 (0.994, 1.002) | 0.997 (0.993, 1.001) | 1.005 (1.001, 1.009)* | 1.003 (0.998, 1.007) |
| Control measure effect |  |  | 0.806 (0.792, 0.821)** |  | 0.810 (0.795, 0.824)** |
| Time trend | 0.993 (0.992, 0.994)** | 0.994 (0.993, 0.995)** | 1.219 (1.198, 1.240)** | 0.993 (0.992, 0.994)** | 1.214 (1.193, 1.235)** |
| χ^2^/*df* | 0.36 | 0.32 | 0.16 | 0.30 | 0.17 |
| *R^2^_fixed_* | 8.34% | 22.1% | 42.1% | 24.8% | 41.9% |
| *R^2^_random_* | 20.0% | 23.3% | 14.0% | 22.7% | 12.8% |
| *∆R^2^_fixed_* | - | 13.8% | 33.7% | 16.4% | 33.6% |

Note: M1, Model with time only; M3 (lag=3 days), Model with city-specific characteristics, meteorological factors at a 3-day lag, and time; M5 (full model, lag=3 days), Model with city-specific characteristics, meteorological factors at a 3-day lag, control measure variable, and time; M3 (lag=7 days), Model with city-specific characteristics, meteorological factors at a 7-day lag, and time; M5 (full model, lag=7 days), Model with city-specific characteristics, meteorological factors at a 7-day lag, control measure variable, and time

RR, Relative risk in incidence rate of COVID-19 for each unit change of variable; χ^2^/*df*, chi-square statistics divided by the degree of freedom; *R^2^_fixed_*, Proportion of variance in the incidence rate (per million population) explained by the fixed effect terms; *R^2^_random_*, Proportion of variance explained by the random effect term of cities’ heterogeneity. *∆R^2^_fixed_*, *R^2^_fixed_* of each model minus *R^2^_fixed_* of M1.

**Discussion**

Although laboratory findings showed that the stability of SARS-CoV-2 was sensitive to temperature and relative humidity [6,25,26] in controlled environments, the meteorological effect varies greatly at population level when host factors were taken into account. In this study, we employed data from 102 Chinese cities which experienced the first wave of epidemic to assess how much variability in COVID-19 activity was attributable to city-level socio-demographic characteristics, meteorological factors, and control measures of level I responses. According to our results, despite a significant association was observed between temperature and risk of COVID-19, very limited variability of the COVID-19 incidence was attributable to meteorological factors once the effect of control measures of level I response was taken into account. Instead, the implementation of control measures was associated with a larger proportion of variance explained with regard to the activity of COVID-19 and the results were robust to variations in lag effects of meteorological factors.

Our findings support that control measures have significant effects on COVID-19 incidences while climatic conditions are less important in the limits of this study. This corroborates with an investigation by te Beest and colleagues [27] which focused on seasonal influenza, another respiratory disease with likely identical transmission route (via contact, droplets, and fomites). They [27] showed that the effect of absolute humidity could only explain very limited proportion of variance in disease transmission intensity, instead, depletion of susceptible during an epidemic that might be done by vaccination contributed to one-third of total variance. Our study suggests a similar perspective that the effect of host factors likely contributes much variability to COVID-19 transmission at population level even though laboratory findings suggested the viral spreading ability of coronavirus reduced in hot condition [28]. This, on the other hand, suggests that if a vaccine is not available, non-pharmaceutical interventions to reduce the frequency of host contacts (e.g. social distancing) are required to induce a decrease in COVID-19 incidence .

Although meteorological factors only explained a small proportion of variance, temperature was negatively associated with the risk of COVID-19 incidence. This is consistent with a study indicating that higher temperature was significantly associated with a linearly decreasing risk of COVID-19 in the United States [7] even though the epidemic has shifted to some very hot areas including Florida, Texas, and Arizona. Another investigation also indicated that low temperature and low absolute humidity were correlated with more COVID-19 deaths in Wuhan [29]. Nevertheless, the association identified in our study contradicts the results of an earlier study showing that high temperature favoured the transmission of COVID-19 in Brazil [30]. Yet, we noted that the Brazil study did not account for the increase in intensity of control measures along time. Such inconsistency of association between temperature and disease transmission was typically observed in respiratory diseases across different zones [14]. While relative humidity has been shown to be correlated with the activity of COVID-19 [7,30], the association is insignificant in our study. Instead, a lower absolute humidity was found to favour COVID-19 activity which may suggest COVID-19 shares similar viral characteristics with influenza in which lower absolute humidity could enhance the survival and transmission of the virus [18].

In our analysis, we employed random effects to capture the city-specific heterogeneity that cannot be accounted for by our data. Random effects term, together with the fixed effects terms, helped to increase the variance explained by the models to around half of the total variability. The remaining unexplained variance could be attributed to many other factors. For example, we did not capture the between-province heterogeneity which might be inherited from the variation in the implementation of level I response and control measures (S1 Table). Different levels of reporting rates may also contribute to the unexplained variance, especially when the public awareness of the newly emerged COVID-19 has increased compared to the start of the epidemic which might only be partly captured by the time effect.

There are several major limitations in our study. First, we did not account for the changes in number of susceptibles (patients?) by taking it as one of the fixed effects in our statistical models. However, given COVID-19 is a newly emerged infectious disease, the effect of variation in number of susceptibles on our results is likely to be comparatively minor [27]. Second, we did not study the impacts of other meteorological variables such as rainfall because majority of studies have only documented the impacts of temperature and humidity. Ultraviolet radiation was also shown to be insignificantly associated with the transmission of COVID-19 [10]. Moreover, since the pollution level in China was likely to be reduced at the moment due to shutdown of business and industrial activities, pollutants were not included in our analysis so as to avoid interpretation of non-causal relationship [31]. However, we cannot completely rule out a potential effect of pollutants on exacerbating the prognosis of COVID-19 especially in elderly with chronic conditions as we observed more cases and deaths in the elderly [32-34]. Third, we used a single variable to capture the effect of control measures of level I response in the model due to complexity in differentiating the impacts of each control measure. Further modelling investigation using more information to rank the importance of factors in explaining the reduction of COVID-19 incidence is warranted.

In conclusion, we showed that even though ambient temperature was associated with COVID-19, the impact of meteorological factors was very limited and only the effect of control measures could explain a large portion of variability in COVID-19 activity. Therefore, we argue stringent control measures are necessary to control COVID-19 regardless the meteorological conditions of an area. Given that no vaccine is available to date, our investigation provides an additional evidence, as advocated by World Meteorological Organization [35], rather than relying on changes in the natural environment for mitigation, active non-pharmaceutical interventions are necessary to curb the COVID-19 pandemic.

**Acknowledgements**

We thank Dr. Tsz Cheung Lee from the Hong Kong Observatory for his assistance in the interpretation of study findings. The work is supported by National Natural Science Foundation of China (31871340, 71974165) and partially supported by Health and Medical Research Fund (INF-CUHK-1, 19181132). The funding organizations had no role in the design and conduct of the study; collection, management, analysis, and interpretation of the data; preparation, review, or approval of the manuscript; and decision to submit the manuscript for publication.

**References**

1. Sohrabi C, Alsafi Z, O'Neill N, et al. World Health Organization declares global emergency: A review of the 2019 novel coronavirus (COVID-19). *Int J Surg*. 2020;76:71-76.
2. Xinhua News Agency [Internet]. *National Emergency Plan for Public Health Emergencies. [in Chinese]* 2020. Available from: <http://www.gov.cn/yjgl/2006-02/26/content_211654.htm> [accessed 31 July 2020]
3. World Health Organisation (WHO). *Coronavirus Disease 2019 (COVID-19) Situation Report – 51.* 2020. Available from: <https://apps.who.int/iris/bitstream/handle/10665/331475/nCoVsitrep11Mar2020-eng.pdf?sequence=1&isAllowed=y> [accessed 31 July 2020]
4. World Health Organisation (WHO). *WHO Director-General's opening remarks at the media briefing on COVID-19 - 11 March 2020.* 2020. Available from: <https://www.who.int/dg/speeches/detail/who-director-general-s-opening-remarks-at-the-media-briefing-on-covid-19---11-march-2020> [accessed 31 July 2020]
5. Lowen AC, Steel J. Roles of humidity and temperature in shaping influenza seasonality. *J Virol*. 2014;88(14):7692-7695.
6. Chin AWH, Chu JTS, Perera MRA, et al. Stability of SARS-CoV-2 in different environmental conditions. *Lancet.* 2020 April 02.
7. Chien LC, Chen LW. Meteorological impacts on the incidence of COVID-19 in the US. *Stoch Env Res Risk A.* 2020:1-6.
8. Ran J, Zhao S, Han L, et al. A Re-analysis in Exploring the Association between Temperature and COVID-19 Transmissibility: An Ecological Study with 154 Chinese Cities. *Eur Respir J*. 2020;2001253.
9. Wu Y, Jing W, Liu J, et al. Effects of temperature and humidity on the daily new cases and new deaths of COVID-19 in 166 countries. *Sci Total Environ*. 2020;729:139051.
10. Yao Y, Pan J, Liu Z, et al. No association of COVID-19 transmission with temperature or UV radiation in Chinese cities. *Eur Respir J*. 2020;55(5):2000517.
11. Xie J, Zhu Y. Association between ambient temperature and COVID-19 infection in 122 cities from China. *Sci Total Environ*. 2020;724:138201.
12. Shen X, Cai C, Li H. Socioeconomic Restrictions Slowdown COVID-19 Far More Effectively than Favorable Weather: Satellite Reveals How Much the World Has Done. *Lancet.* 2020 May 28. Available at SSRN: <https://ssrn.com/abstract=3616014>. (Preprint)
13. National Health Commissions (NHC) of the People's Republic of China. *Epidemic situation update.* 2020. Available at: <http://www.nhc.gov.cn/xcs/yqtb/list_gzbd.shtml> [accessed 31 July 2020]
14. Zhang J, Litvinova M, Wang W, et al. Evolving epidemiology and transmission dynamics of coronavirus disease 2019 outside Hubei province, China: a descriptive and modelling study. *Lancet Infect Dis*. 2020;20(7):793-802.
15. National Climate Data Center (NCDC). 2020. Available at: <https://www.ncdc.noaa.gov/> [accessed 31 July 2020]
16. Chong KC, Lee TC, Bialasiewicz S, et al. Association between meteorological variations and activities of influenza A and B across different climate zones: a multi-region modelling analysis across the globe. *J Infect*. 2020;80(1):84-98.
17. Chong KC, Liang J, Jia KM, et al. Latitudes mediate the association between influenza activity and meteorological factors: A nationwide modelling analysis in 45 Japanese prefectures from 2000 to 2018. *Sci Total Environ*. 2020;703:134727.
18. Shaman J, Kohn M. Absolute humidity modulates influenza survival, transmission, and seasonality. *Proc Natl Acad Sci U S A*. 2009;106(9):3243-3248.
19. Han L, Ran J, Chan KH, et al. Indoor environmental factors and acute respiratory illness in a prospective cohort of community-dwelling older adults. *J Infect Dis*. 2020;jiaa188.
20. Chong KC, Goggins W, Zee BC, Wang MH. Identifying meteorological drivers for the seasonal variations of influenza infections in a subtropical city – Hong Kong. *Int J Environ Res Public Health*. 2015;12(2):1560-1576.
21. Loh TP, Lai FY, Tan ES, et al. Correlations between clinical illness, respiratory virus infections and climate factors in a tropical paediatric population. *Epidemiol Infect*. 2011;139(12):1884-1894.
22. Tetens O. Über einige meteorologische Begriffe [In German]. Z. Geophys. 1930;6:297-309.
23. National Bureau of Statistics of China (NBSC). *China Statistical Yearbook.* 2020 Available at: <http://www.stats.gov.cn/tjsj/ndsj/2019/indexeh.htm> [accessed 31 July 2020]
24. Nakagawa S, Johnson PCD, Schielzeth H. The coefficient of determination *R*^2^ and intra-class correlation coefficient from generalized linear mixed-effects models revisited and expanded. *J R Soc Interface*. 2017;14(134):20170213.
25. van Doremalen N, Bushmaker T, Morris DH, et al. Aerosol and Surface Stability of SARS-CoV-2 as Compared with SARS-CoV-1. *N Engl J Med*. 2020;382(16):1564-1567.
26. Biryukov J, Boydston JA, Dunning RA, et al. Increasing Temperature and Relative Humidity Accelerates Inactivation of SARS-CoV-2 on Surfaces. *mSphere*. 2020;5(4):e00441-20.
27. te Beest DE, van Boven M, Hooiveld M, van den Dool C, Wallinga J. Driving factors of influenza transmission in the Netherlands. *Am J Epidemiol*. 2013;178(9):1469-1477.
28. Chan KH, Peiris JS, Lam SY, Poon LL, Yuen KY, Seto WH. The Effects of Temperature and Relative Humidity on the Viability of the SARS Coronavirus. *Adv Virol*. 2011;2011:734690.
29. Ma Y, Zhao Y, Liu J, et al. Effects of temperature variation and humidity on the death of COVID-19 in Wuhan, China. *Sci Total Environ*. 2020;724:138226.
30. Auler AC, Cássaro FAM, da Silva VO, Pires LF. Evidence that high temperatures and intermediate relative humidity might favor the spread of COVID-19 in tropical climate: A case study for the most affected Brazilian cities. *Sci Total Environ*. 2020;729:139090.
31. Zhao S. To avoid the noncausal association between environmental factor and COVID-19 when using aggregated data: Simulation-based counterexamples for demonstration. *Sci Total Environ*. 2020;141590.
32. Ran J, Zhao S, Han L, et al. The ambient ozone and COVID-19 transmissibility in China: A data-driven ecological study of 154 cities. *J Infect*. 2020;81(3):e9-e11.
33. Yao Y, Pan J, Liu Z, et al. Temporal association between particulate matter pollution and case fatality rate of COVID-19 in Wuhan. *Environ Res*. 2020;189:109941.
34. Yao Y, Pan J, Wang W, et al. Association of particulate matter pollution and case fatality rate of COVID-19 in 49 Chinese cities. *Sci Total Environ*. 2020;741:140396.
35. World Meteorological Organization. Health: Correlations? Busting the myths. 2020 Available at: <https://public.wmo.int/en/resources/coronavirus-covid-19/impacts-public-health> [accessed 31 July 2020]

**Supporting information**

**S1 Table. Summary of control measures of Level I response in 27 provinces/municipalities**

**S2 Table. Changes of R-square and relative risks (95% confidence intervals) of the variables when interaction terms were included**
