# Supplementary material for: Limited role for meteorological factors on the variability in COVID-19 incidence: A retrospective study of 102 Chinese cities
Source: PLoS Negl Trop Dis. 2021 Feb 24;15(2):e0009056. doi: 10.1371/journal.pntd.0009056 (PMC7904227; doi:10.1371/journal.pntd.0009056)
Supplement: Attachment — Submitted filename: Response to reviewers.docx [file pntd.0009056.s007.docx]

**Response to Reviewers’ Comments**

PNTD-D-20-01614

Limited role for meteorological factors on the variability in COVID-19 incidence: A retrospective study of 102 Chinese cities

Chong et al.

**Comments from the reviewers:**

**Reviewer #1:**

1. I wonder if the procedure for incorporating the incremental effect of control measures of level I responses in the statistical model does not exaggerate this factor too much. Xmij in Equation 3 grows more and more positive after the date of control measure implementation. This way possible temporal variability in the implementation of the measures is not considered.

**Response**: Thanks for the comment. The idea to incorporate both Xmij and day-after-intervention variable in the model is due to the fact that the effect of control measures should have impact on the incidence rate by time (instead of an average post-intervention effect), which follows the concept of segmented linear regression (Wagner et al. 2002). As we can see in Figure 2D, the epidemic tends entered a descending phase after the implementation of control measures. If we do not model the effect of control measures incrementally, for example, coding the effect as dummy variable (Xmij=1 after the date of implementation), would result in a worse model fit. Indeed, we have compared the model fitness between assuming the effect being incremental and a constant effect. All fitness statistics show the incremental effect assumption allows a better fit of data i.e. Bayesian information criterion (BIC)=4642 for assuming an incremental effect vs BIC=5197 assuming a constant effect, whereas Akaike information criterion (AIC)=4613 for assuming an incremental effect vs AIC=5168 assuming a constant effect.

Reference:

Wagner AK, Soumerai SB, Zhang F, Ross‐Degnan D. Segmented regression analysis of interrupted time series studies in medication use research. Journal of clinical pharmacy and therapeutics. 2002 Aug;27(4):299-309..

2. On the city-specific random effect: differences in local measures and the way they are applied are very important and must be accounted for somehow in this random effect. How is this done in this research? Please elaborate on this. How sensitive is this choice for the overall result?

**Response**: Thanks for the comment. City specific random effect was used to account for the between-city heterogeneity that may likely be affecting the COVID-19 incidence and was unable to be collected for model fitting, despite we have accounted for some other city-specific variables such as population density and GDP per capita. We acknowledge the random effect in our model setting could not account for the variability in local measures since the effect started only after the date of control measure implementation (i.e. Xmij>0).

However, we agree that the differences in local measures could affect the sensitivity of the results so we have additionally performed an analysis which includes each control measure by categorizing them into different types (i.e. Social distancing measures, screening and contact tracing, quarantine of risky populations, hospital-related measures, and other public health measures). A similar model form was used (please see the revised S1 Table and S1 Appendix) and the results were as follow:

**Table 5**. Comparison of changes in R-square among different models and relative risks (95% confidence intervals) of the variables with control measures stratified by type

| Variables | M4 | M5 (full model) |
| --- | --- | --- |
| City-specific characteristics |  |  |
| Population density (in /100 km^2^) | 1.017 (0.990, 1.044) | 1.022 (0.994, 1.051) |
| GDP per capita (in 10,000 Chinese Yuan) | 1.031 (0.970, 1.095) | 1.040 (0.974, 1.109) |
| Proportion of tertiary education (in %) | 0.984 (0.941, 1.030) | 0.945 (0.929, 1.023) |
| Proportion of elderly population (in %) | 0.951 (0.845, 1.069) | 0.921 (0.812, 1.045) |
| Distances to Wuhan (in 100 km) | 1.009 (0.979, 1.040) | 0.985 (0.952, 1.019) |
| Meteorological factors |  |  |
| Temperature (in ^o^C) |  | 0.970 (0.955, 0.986)* |
| Relative humidity (in %) |  | 0.993 (0.989, 0.997)* |
| Control measure effect |  |  |
| Social distancing | 0.915 (0.896, 0.935)** | 0.912 (0.892, 0.932)** |
| Screening and contact tracing | 0.942 (0.923, 0.960)** | 0.945 (0.926, 0.965)** |
| Quarantine of risky populations | 1.007 (0.993, 1.022) | 1.009 (0.994, 1.024) |
| Hospital-related measures | 0.954 (0.942, 0.967)** | 0.954 (0.941, 0.967)** |
| Other public health measures | 0.942 (0.927, 0.957)** | 0.942 (0.927, 0.958)** |
| Time trend | 1.161 (1.146, 1.175)** | 1.161 (1.146, 1.176)** |
| χ^2^/*df* | 0.16 | 0.15 |
| *R^2^_fixed_* | 37.0% | 38.7% |
| *R^2^_random_* | 19.2% | 20.5% |
| *∆R^2^_fixed_* | 36.0% | 37.7% |

Note: M4, Model with city-specific characteristics, different control measures, and time; M5, Model with city-specific characteristics, meteorological factors, different control measures, and time (full model). RR, Relative risk in incidence rate of COVID-19 for each unit change of variable; χ^2^/*df*, chi-square statistics divided by the degree of freedom; *R^2^_fixed_*, Proportion of variance in the incidence rate (per million population) explained by the fixed effect terms; *R^2^_random_*, Proportion of variance explained by the random effect term of cities’ heterogeneity. *∆R^2^_fixed_*, *R^2^_fixed_* of each model minus *R^2^_fixed_* of M1 in Table 1.

**p*<0.05; ***p*<0.001

According to the results, the overall variance explained in the models was reduced by around 8% when the control measures were categorized by types. However, as with the major finding, including the meteorological effects in the model only resulted in 2% increase in the explained variance with the statistical significances of the temperature and relative humidity remained unchanged. Among the control measure types, imposing social distancing, screening and contact tracing, hospital-related measures, and other public health measures were significantly associated with a lower risk of COVID-19. Quarantine of risky populations was not found to be a significant predictor.

We have revised the methods, results, and discussion for this additional analysis:

“*Apart from that, we categorized the control measures into 5 types: social distancing, screening and contact tracing, quarantine of risky populations, hospital-related measures, and other public health measures in order to examine the robustness of the composite variable of the level I responses in the GLMM and to assess the statistical significance for each types of the control measures. A similar model form was used (S1 Appendix).*” (Track change: Paragraph 4 in Statistical analysis)

“*When the control measures were categorized by type, the overall variance explained in the models was reduced by around 8% (Table 5). However, as with the major finding, including the meteorological effects in the model (M5) only resulted in 2% increase in the explained variance with the statistical significances of the temperature and relative humidity remained unchanged. Among the control measure types, imposing social distancing (RR=0.912, 95% CI: 0.892-0.932, p<0.001), screening and contact tracing (RR=0.945, 95% CI: 0.926-0.965, p<0.001), hospital-related measures (RR=0.954, 95% CI: 0.941-0.967, p<0.001), and other public health measures (RR=0.942, 95% CI: 0.927-0.958, p<0.001) were significantly associated with a lower risk of COVID-19. Quarantine of risky populations was not found to be a significant predictor.*” (Track change: Paragraph 7 in Results)

“…*For example, we did not capture the between-province heterogeneity which might be inherited from the variation in the compliance of the control measures in level I response (S1 Table). Nevertheless, we conducted an additional analysis by including different types of control measures in the GLMMs and the results were consistent with our major finding though a decrease of model fitness was observed. We also found a majority of control measures (i.e. social distancing, screening and contact tracing, hospital-related measures, and other public health measures) was significantly associated with a lower risk of COVID-19 activity*.” (Track change: Paragraph 4 in Discussion)

3. Experiences show that the day of the week affects data collection. At some days in the week people tend to visit the doctor more frequently for testing than on other days. So why is this variable not considered in the statistical model?

**Response**: Thanks for your suggestion. We agree that in typical time series studies that the outcome is not in acute condition such as some seasonal infectious diseases (Cheng at al. 2020) and chronic diseases (Mohammad et al. 2020), the day-of-week variable likely affect behavior of consultation as well as confounding the risk of health outcome. However, for COVID-19 that is newly emerged into the community, we do not expect the day-of-week pattern would affect the visiting behavior given a high awareness for the related symptoms and intense surveillance from healthcare officials. We have done a sensitivity analysis by adding the day-of-week term into the full model and the result is as follow:

**S4 Table**. Changes of R-square and relative risks (95% confidence intervals) of the variables when day-of-week was included

| Variables | Relative risks (95% confidence intervals) |
| --- | --- |
| City-specific characteristics |  |
| Population density (in /100 km^2^) | 1.019 (0.995-1.047) |
| GDP per capita (in 10,000 Chinese Yuan) | 1.021 (0.966-1.078) |
| Proportion of tertiary education (in %) | 1.000 (0.961-1.042) |
| Proportion of elderly population (in %) | 0.907 (0.815-1.010) |
| Distances to Wuhan (in 100 km) | 0.987 (0.958-1.016) |
| Meteorological factors |  |
| Temperature (in ^o^C) | 0.985 (0.969-0.999)* |
| Relative humidity (in %) | 0.993 (0.988-0.997)* |
| Control measure effect | 0.754 (0.738-0.771)** |
| Day of Week | 0.989 (0.964-1.015) |
| Time trend | 1.227 (1.206-1.248)** |
| χ^2^/*df* | 0.11 |
| *R^2^_fixed_* | 45.8% |
| *R^2^_random_* | 13.9% |
| *∆R^2^_fixed_* | 44.8% |

RR: Relative risk in incidence rate of COVID-19 for each unit change of variable; χ^2^/*df*: chi-square statistics divided by the degree of freedom; *R^2^_fixed_*: Proportion of variance in the incidence rate (per million population) explained by the fixed effect terms; *R^2^_random_*: Proportion of variance explained the random effect term of cities’ heterogeneity. *∆R^2^_fixed_*: *R^2^_fixed_* of each model minus *R^2^_fixed_* of M1.

**p*-value<0.05; ** *p*-value<0.001

The result shows that not just day-of-week is statistically insignificant, the effects of other variables as well as the R-squares only changed slightly. We have included this additional analysis into our revised manuscript.

Reference:

Cheng W, Li H, Zhang X, Sun W, Chong KC, Lau SY, Yu Z, Liu S, Ling F, Pan J, Chen E. The association between ambient particulate matters, nitrogen dioxide, and childhood scarlet fever in Hangzhou, Eastern China, 2014–2018. Chemosphere. 2020.1;246:125826.

Mohammad KN, Chan EY, Wong MC, Goggins WB, Chong KC. Ambient temperature, seasonal influenza and risk of cardiovascular disease in a subtropical area in Southern China. Environmental Research. 2020.18:109546.

4. Explain the ** in the tables; Are the boxplots showing the max/min values or the 95/5% values?

**Response**: Apologies for the missing footnote. The * and ** indicate p-value <0.05 and <0.001 respectively. We have added the footnotes in the tables.

5. The conclusion of this manuscript is perhaps not that meteorology has few impact on COVID19, but that the impact of meteorology could not be assessed due to the huge impact of the control measures, which is clearly suggested in the title (fine for me!), but some conclusions should be weakened more in the manuscript.

**Response**: Thanks for the suggestion and we have revised some parts in the discussion to ensure the message conveyed is correct. For example:

“*According to our results, despite temperature and relative humidity were significantly associated with the risk of COVID-19, we could not identify a substantial variability of the COVID-19 incidence was attributable to meteorological factors once the effect of control measures of level I response was taken into account…*” (Track change: Paragraph 1 in Discussion)

“*Although we could not show the meteorological factors explained a large proportion of variance…*” (Track change: Paragraph 3 in Discussion)

“*In conclusion, even though the meteorological factors were associated with COVID-19, we could not find an apparent impact of them and only the effect of control measures could explain a large portion of variability in COVID-19 activity.*” (Track change: Paragraph 1 in Discussion)

6. Overall an interesting and relevant paper, covering a contemporary topic. The manuscript is fairly well written, and the applied basic statistical methodology seems to be sound enough.

**Response**: Thanks for the positive comment. (Track change: Paragraph 6 in Discussion)

7. My two main concerns are that these data sets only cover a very short period so that the effect of meteorological factors cannot be properly identified since the meteorological variation seems not very large (based on what I could see from the graphs in Fig 2), and since the control measures that were taken are so draconic that these eliminate all other possible factors. Meteorological factors affecting COVID19 activity are just drawn by the control measures. Let me be clear, I am not judging the control measures that were taken.

**Response**: Sorry for the presentation of figure 2, which may be misleading. If we look at all the Chinese cities included, daily temperature, relative humidity, and vapour pressure respectively span the range of -23.6^o^C to 29.5^o^C, 9.4% to 100%, and 0.56hPa to 29.9hPa. The ranges indeed covered majority of meteorological variation in most Chinese cities across a year though we acknowledge the extreme cold and heat conditions are unable to be studied. We have revised Table 1 and included the quantile statistics for the meteorological factors:

**Table 1**. Descriptive statistics of city-specific characteristics and meteorological factors in 102 cities.

|  | Minimum | 25^th^ percentile | Median | 75^th^ percentile | Maximum |
| --- | --- | --- | --- | --- | --- |
| Population (in 10,000) | 60 | 413 | 605 | 825 | 3,397 |
| Population density (in /km^2^) | 66 | 254 | 514 | 706 | 6,523 |
| GDP per capita (in 10,000 Chinese Yuan) | 2.2 | 3.9 | 6.3 | 8.8 | 19.0 |
| Proportion of individuals having tertiary education (%) | 8.5 | 10.9 | 12.0 | 13.7 | 42.3 |
| Proportion of elderly population (%) | 7.4 | 9.5 | 10.8 | 11.7 | 12.9 |
| Distances to Wuhan (in 100 km) | 2.1 | 6.7 | 9.3 | 12.5 | 32.7 |
| Ambient temperature over all cities (^o^C) | -23.6 | 3.6 | 8.8 | 13.7 | 29.5 |
| Relative humidity over all cities (%) | 9.4 | 58.4 | 72.8 | 84.2 | 100.0 |
| Vapour pressure over all cities (hPa) | 0.6 | 4.9 | 7.9 | 11.3 | 29.9 |

The results section has described the results:

“*Across all the included cities, the daily ambient temperature and relative humidity ranged from -23.6^o^C to 29.5^o^C and 9.4% to 100% respectively (Table 1). The overall median of city-specific mean temperature was 6.9^o^C (range: -15.0^o^C to 22.6^o^C) and the median of city-specific mean temperature increased from 4.8^o^C (range: -18.5^o^C to 22.2^o^C) in January 2020 to 10.0^o^C (range: -7.4^o^C to 24.3^o^C) in March 2020 (Fig 2A). The overall median of city-specific mean relative humidity was 74.4% (Range: 44.9% to 89.7%) and the median of city-specific mean relative humidity decreased slightly from 76.3% (range: 51.6% to 90.9%) in January 2020 to 73.8% (range: 30.3% to 97.9%) in March 2020 (Fig 2B).*” (Track change: Paragraph 2 in Results)

We also discuss this concern in the discussion section:

“…*In addition, our study period covered the first wave of COVID-19 epidemic which only lasted for three months. Our findings may thus not be generalized in other seasons although our study period covered a wide range of meteorological variation in most Chinese cities across a year…”* (Track change: Paragraph 5 in Discussion)

**Reviewer #2:**

1. The authors present the results of an analysis on the determinants of COVID-19 over more than 100 Chinese cities. The tested variables belong to three major groups: socio-demographic aspects, meteorology and control measures. The study is relevant as it contributes to the current global efforts towards understanding the drivers of the spread of the virus. Therefore, I would recommend its publication provided a number of remarks/questions are clarified. Especially the remarks 2 to 6 concern aspects of the study that – I consider- deserve more elaboration or argumentation from the authors.

**Response**: Thank you for the positive comment and we have addressed your comments accordingly.

2. The caption of Figure 1 is “The 102 Chinese cities selected in the study”. The Figure, however, seems to represent municipalities or provinces instead of cities. Is that the case? Are the colored shapes in the map actually showing the 27 provinces (Line 116) where the study was conducted? Moreover, the Figure intends to represent a map but no geographical references (like grid with coordinates, North arrow, scale) are given. It would also be useful to show labels indicating the location of (at least) the main places mentioned in the manuscript (Hubei, Ningxia, Guangdong, etc). In its current state the Figure 1 is not really helpful to the reader who is not familiar with the geography in that part of the world. I would also recommend to write the full word ‘Figure’ instead of ‘Fig’ in the body of the text and the figure caption.

**Response**: Thanks for pointing it out and we apologize for the mistake. The figure has been revised and the cities have been pinpointed in the new map. Some major cities have also been labelled. The use of ‘Fig’ instead of ‘Figure’ is owing to the format requirement in PLOS journal series (https://journals.plos.org/plosntds/s/submission-guidelines#loc-style-and-format).

3. Equation 2 indicates that the time step of the analysis is 1 day. However, the variables in the model related to city-specific characteristics (population density, GDP, distance to Wuhan, etc) do not change daily, by nature; in fact, they remain unchanged during the studied period. For each particular city they behave as constants in the model as compared to other time-varying potential drivers like meteorology and control measure effects. What can be the impact of combining time-varying and constant drivers of the spread in the model of Equation 2. Was that a good approach? Could that be the explanation of the small difference between the results of M1 and those of M2, as explained in Lines 227-228? Could you elaborate more on that?

**Response**: Thanks for pointing it out. As what you indicated, while city-specific characteristics vary between cities (not constant) and the meteorological factors are time-dependent, if we model these variables of different scales using traditional approaches (e.g. multiple linear regressions), the effect of meteorological factors will likely be overestimated owing to the intra-city variability from its scale. Models with both time-variant and time-invariant covariates are not uncommon in literature and reference books of GLMM (for example, page 414 - 421 of Fitzmaurice et al. 2011 and page 247- 253 of Diggle et al. 2002). The current setting is analogous to a longitudinal study of RCT, in which the multiple measurements of a subject are modelled with characteristics of a study that do not change with time, for example, gender and treatment groups, and other time-variant covariates.

Because of this reason, in this study we analyzed the data using generalized linear mixed effect models (GLMMs) in order to account for the effect of variables from different levels. To be honest, it is hard to say whether this is the best approach as there are some other statistical approaches that can be used to deal with multi-level data such as generalized estimating equations. However, at least the full model of our GLMM using both fixed and random effects could account for ~60% of variance in the data and should thus be a promising tool for comparing the contribution of the independent effects.

Instead of the structure of data, we believe the small difference between the results of M1 and those of M2 is due to the limited number of city-specific variables that cannot explain the incidence curve. For example, we expected the city-specific testing rate, healthcare capacity, and medical manpower should be more correlated with the disease spread in general. However, without available data, just few demographic and socioeconomic metrics could not explain much for the epidemic even though the random effects captured a proportion of city-specific heterogeneity.

Reference:

Diggle, P., Diggle, P. J., Heagerty, P., Liang, K. Y., Heagerty, P. J., & Zeger, S. (2002). Analysis of longitudinal data. Oxford University Press.

Fitzmaurice, G. M., Laird, N. M., & Ware, J. H. (2012). Applied longitudinal analysis (Vol. 998). John Wiley & Sons.

4. Lines 165-166 explain the role of the timei variable. This variable seems to be quite arbitrary and only dependent on the date chosen by the researchers to start the study. Therefore, not really independent. How can this decision be justified? And what about the collinearity between the timei and the xmij variable (Equation 3)? I guess the value of those variables is quite similar as they are built on the same manner: starting from 0 and adding 1 every elapsed day.

**Response**: Thanks for the comment. The time trend variable (timei) is a proxy variable that captures the undefined variable along time, for example, the decrease of susceptible individuals. The term is very common to be used in similar types of time series analysis for an adjustment of the overall trend of disease pattern (Chong et al. 2020). The original study period was chosen based on the date range that can cover the epidemic of all the cities. Nevertheless, we agree that the date chosen by us may be a bit subjective (thanks for pointing it out!) and we have thus completely re-conducted our analysis using only the period from the start of epidemic (i.e. date of having the first case with his illness onset) to the end of epidemic (i.e. date of the last case) in each of the included cities. We found the model fitness has been improved in terms of Bayesian information criterion and Akaike information criterion and the general findings in the study remains unchanged.

Typically, even the time trend exhibits a certain correlation (not very strong) with other variables, it is suggested to be kept in the model. Nevertheless, we have checked the variance inflation factor (VIF) of the time trend in our revised analysis and the VIF is <10, indicating a not very strong multicollinearity. Also, we have tried to exclude the term of time trend in the model and the R-square contributed by the meteorological factors is just <5%, which is consistent with the results in Table 2.

Reference:

Chong KC, Liang J, Jia KM, Kobayashi N, Wang MH, Wei L, Lau SY, Sumi A. Latitudes mediate the association between influenza activity and meteorological factors: A nationwide modelling analysis in 45 Japanese prefectures from 2000 to 2018. Science of The Total Environment. 2020 Feb 10;703:134727.

5. The values of the variable xmij as indicated in Equation 3 are only dependent on the date of implementation of the control measures. Does it mean an implicit assumption that the impact of the different measures listed in Table S1 is equivalent (i.e. the efficiency of the control measures in the 27 provinces is comparable)?

**Response**: Thanks for the comment and we assumed the impact of the measures are equivalent to that in the original manuscript. Nevertheless, echoed with a concern from another reviewer, we agree that the differences in local measures could affect the sensitivity of the results (though compliance was hard to be quantified). Hence, we have additionally performed an analysis which included each control measure by categorizing them into different types (i.e. Social distancing measures, screening and contact tracing, quarantine of risky populations, hospital-related measures, and other public health measures). A similar model form was used (please see the revised S1 Table and S1 Appendix) and the results were as follow:

**Table 5**. Comparison of changes in R-square among different models and relative risks (95% confidence intervals) of the variables with control measures stratified by type

| Variables | M4 | M5 (full model) |
| --- | --- | --- |
| City-specific characteristics |  |  |
| Population density (in /100 km^2^) | 1.017 (0.990, 1.044) | 1.022 (0.994, 1.051) |
| GDP per capita (in 10,000 Chinese Yuan) | 1.031 (0.970, 1.095) | 1.040 (0.974, 1.109) |
| Proportion of tertiary education (in %) | 0.984 (0.941, 1.030) | 0.945 (0.929, 1.023) |
| Proportion of elderly population (in %) | 0.951 (0.845, 1.069) | 0.921 (0.812, 1.045) |
| Distances to Wuhan (in 100 km) | 1.009 (0.979, 1.040) | 0.985 (0.952, 1.019) |
| Meteorological factors |  |  |
| Temperature (in ^o^C) |  | 0.970 (0.955, 0.986)* |
| Relative humidity (in %) |  | 0.993 (0.989, 0.997)* |
| Control measure effect |  |  |
| Social distancing | 0.915 (0.896, 0.935)** | 0.912 (0.892, 0.932)** |
| Screening and contact tracing | 0.942 (0.923, 0.960)** | 0.945 (0.926, 0.965)** |
| Quarantine of risky populations | 1.007 (0.993, 1.022) | 1.009 (0.994, 1.024) |
| Hospital-related measures | 0.954 (0.942, 0.967)** | 0.954 (0.941, 0.967)** |
| Other public health measures | 0.942 (0.927, 0.957)** | 0.942 (0.927, 0.958)** |
| Time trend | 1.161 (1.146, 1.175)** | 1.161 (1.146, 1.176)** |
| χ^2^/*df* | 0.16 | 0.15 |
| *R^2^_fixed_* | 37.0% | 38.7% |
| *R^2^_random_* | 19.2% | 20.5% |
| *∆R^2^_fixed_* | 36.0% | 37.7% |

Note: M4, Model with city-specific characteristics, different control measures, and time; M5, Model with city-specific characteristics, meteorological factors, different control measures, and time (full model). RR, Relative risk in incidence rate of COVID-19 for each unit change of variable; χ^2^/*df*, chi-square statistics divided by the degree of freedom; *R^2^_fixed_*, Proportion of variance in the incidence rate (per million population) explained by the fixed effect terms; *R^2^_random_*, Proportion of variance explained by the random effect term of cities’ heterogeneity. *∆R^2^_fixed_*, *R^2^_fixed_* of each model minus *R^2^_fixed_* of M1 in Table 1.

**p*<0.05; ***p*<0.001

According to the results, the overall variance explained in the models was reduced by around 8% when the control measures were categorized by type. However, as with the major finding, including the meteorological effects in the model only resulted in 2% increase in the explained variance with the statistical significances of the temperature and relative humidity remain unchanged. Among the control measure types, imposing social distancing, screening and contact tracing, hospital-related measures, and other public health measures were significantly associated with a lower risk of COVID-19. Quarantine of risky populations was not found to be a significant predictor.

We have revised the methods, results, and discussion for this additional analysis:

“*Apart from that, we categorized the control measures into 5 types: social distancing, screening and contact tracing, quarantine of risky populations, hospital-related measures, and other public health measures in order to examine the robustness of the composite variable of the level I responses in the GLMM and to assess the statistical significance for each types of the control measures. A similar model form was used (S1 Appendix).*” (Track change: Paragraph 4 in Statistical analysis)

“*When the control measures were categorized by type, the overall variance explained in the models was reduced by around 8% (Table 5). However, as with the major finding, including the meteorological effects in the model (M5) only resulted in 2% increase in the explained variance with the statistical significances of the temperature and relative humidity remained unchanged. Among the control measure types, imposing social distancing (RR=0.912, 95% CI: 0.892-0.932, p<0.001), screening and contact tracing (RR=0.945, 95% CI: 0.926-0.965, p<0.001), hospital-related measures (RR=0.954, 95% CI: 0.941-0.967, p<0.001), and other public health measures (RR=0.942, 95% CI: 0.927-0.958, p<0.001) were significantly associated with a lower risk of COVID-19. Quarantine of risky populations was not found to be a significant predictor.*” (Track change: Paragraph 7 in Results)

“…*For example, we did not capture the between-province heterogeneity which might be inherited from the variation in the compliance of the control measures in level I response (S1 Table). Nevertheless, we conducted an additional analysis by including different types of control measures in the GLMMs and the results were consistent with our major finding though a decrease of model fitness was observed. We also found a majority of control measures (i.e. social distancing, screening and contact tracing, hospital-related measures, and other public health measures) was significantly associated with a lower risk of COVID-19 activity*.” (Track change: Paragraph 4 in Discussion)

6. One of the important conclusions of the study is that “the impact of meteorological factors was very limited” (Discussions section). The considered period of the study was, however, quite short and covered only one season; i.e. only a (small) fraction of the annual range of variation of temperature and humidity was investigated. Could we be confident that such a conclusion will hold in other seasons of the year too? If not, it should be mentioned in the text.

**Response**: Thank you for your comment and we apologize for the presentation of figure 2 which may be misleading. If we look at all the Chinese cities included, daily temperature, relative humidity, and vapour pressure respectively span the range of -23.6^o^C to 29.5^o^C, 9.4% to 100%, and 0.56hPa to 29.9hPa. The ranges indeed cover a majority of meteorological variation in most Chinese cities across a year though we acknowledge the extreme cold and heat conditions are unable to be studied. We have revised Table 1 and included the quantile statistics for the meteorological factors:

**Table 1**. Descriptive statistics of city-specific characteristics and meteorological factors in 102 cities.

|  | Minimum | 25^th^ percentile | Median | 75^th^ percentile | Maximum |
| --- | --- | --- | --- | --- | --- |
| Population (in 10,000) | 60 | 413 | 605 | 825 | 3,397 |
| Population density (in /km^2^) | 66 | 254 | 514 | 706 | 6,523 |
| GDP per capita (in 10,000 Chinese Yuan) | 2.2 | 3.9 | 6.3 | 8.8 | 19.0 |
| Proportion of individuals having tertiary education (%) | 8.5 | 10.9 | 12.0 | 13.7 | 42.3 |
| Proportion of elderly population (%) | 7.4 | 9.5 | 10.8 | 11.7 | 12.9 |
| Distances to Wuhan (in 100 km) | 2.1 | 6.7 | 9.3 | 12.5 | 32.7 |
| Ambient temperature over all cities (^o^C) | -23.6 | 3.6 | 8.8 | 13.7 | 29.5 |
| Relative humidity over all cities (%) | 9.4 | 58.4 | 72.8 | 84.2 | 100.0 |
| Vapour pressure over all cities (hPa) | 0.6 | 4.9 | 7.9 | 11.3 | 29.9 |

The results have described the results:

“*Across all the included cities, the daily ambient temperature and relative humidity ranged from -23.6^o^C to 29.5^o^C and 9.4% to 100% respectively (Table 1). The overall median of city-specific mean temperature was 6.9^o^C (range: -15.0^o^C to 22.6^o^C) and the median of city-specific mean temperature increased from 4.8^o^C (range: -18.5^o^C to 22.2^o^C) in January 2020 to 10.0^o^C (range: -7.4^o^C to 24.3^o^C) in March 2020 (Fig 2A). The overall median of city-specific mean relative humidity was 74.4% (Range: 44.9% to 89.7%) and the median of city-specific mean relative humidity decreased slightly from 76.3% (range: 51.6% to 90.9%) in January 2020 to 73.8% (range: 30.3% to 97.9%) in March 2020 (Fig 2B).*” (Track change: Paragraph 2 in Results)

We also discuss this concern in the discussion section:

“…*In addition, our study period covered the first wave of COVID-19 epidemic which only lasted for three months. Our findings may thus not be generalized in other seasons although our study period covered a wide range of meteorological variation in most Chinese cities across a year…”* (Track change: Paragraph 5 in Discussion)

7. If I understood well, the study did not investigate whether the incidence was somehow related to climatic zones; we do not know whether the incidence is higher/lower in dry/humid warm/cold regions. The box plots in Figures 2A, 2B, 2C show, however, that the spatial variability is considerable. Such analysis would strongly support the conclusion stated in the manuscript.

**Response**: Thank you for your suggestion and we have conducted an additional stratified analysis by climate zones. In the new stratified analysis, we examined the difference in proportion of variance explained by factors between temperate and subtropical/tropical cities. Of the 102 included Chinese cities, 45 located in the temperate zone, and 57 located in the subtropical or tropical zones. According to the results, we found the additional variances explained by the control measures were similar between the temperate and subtropical/tropical cities when compared with the variance explained in the model without the effects of control measure. However, interestingly, we found the contribution of meteorological factors in the explained variance of the subtropical/tropical cities was around 3-fold more than that in the temperate cities (i.e. ∆*R^2^_fixed_*=14.4% vs ∆*R^2^_fixed_*=5.04% in M3). The temperature and relative humidity even became statistically insignificant in the full model when fitting the data of temperate cities (temperature: RR=0.981, 95% CI: 0.952-1.010, *p*=0.198; relative humidity: RR=0.997, 95% CI: 0.990-1.004, *p*=0.458). Table 4 has been newly developed to show the statistics:

**Table 4**. Comparison of changes in R-square among different models and relative risks (95% confidence intervals) of the variables by climate zone

| Climate zone | Variables | M1 (null model) | M3 | M5 (full model) |
| --- | --- | --- | --- | --- |
| Temperate | City-specific characteristics |  |  |  |
|  | Population density (in /100 km^2^) |  | 0.971 (0.880, 1.072) | 0.959 (0.854, 1.077) |
|  | GDP per capita (in 10,000 Chinese Yuan) |  | 0.975 (0.904, 1.053) | 0.946 (0.865, 1.034) |
|  | Proportion of tertiary education (in %) |  | 1.031 (0.988, 1.076) | 1.046 (0.994, 1.100) |
|  | Proportion of elderly population (in %) |  | 0.914 (0.787, 1.060) | 0.923 (0.777, 1.097) |
|  | Distances to Wuhan (in 100 km) |  | 0.974 (0.934, 1.016) | 0.987 (0.940, 1.037) |
|  | Meteorological factors |  |  |  |
|  | Temperature (in ^o^C) |  | 0.959 (0.934, 0.984)* | 0.981 (0.952, 1.010) |
|  | Relative humidity (in %) |  | 0.996 (0.990, 1.003) | 0.997 (0.990, 1.004) |
|  | Control measure effect |  |  | 0.771 (0.745, 0.798)** |
|  | Time trend | 1.015 (1.009, 1.021)** | 1.018 (1.011, 1.026)** | 1.219 (1.184, 1.254)** |
|  | χ^2^/*df* | 0.24 | 0.23 | 0.09 |
|  | *R^2^_fixed_* | 2.40% | 7.43% | 43.1% |
|  | *R^2^_random_* | 15.1% | 14.2% | 12.1% |
|  | *∆R^2^_fixed_* | - | 5.04% | 40.7% |
| Subtropical/tropical | City-specific characteristics |  |  |  |

|  | Population density (in /100 km^2^) |  | 1.018 (0.988, 1.048) | 1.014 (0.989, 1.039) |
| --- | --- | --- | --- | --- |
|  | GDP per capita (in 10,000 Chinese Yuan) |  | 1.083 (1.002, 1.170)* | 1.071 (1.004, 1.142)* |
|  | Proportion of tertiary education (in %) |  | 0.957 (0.876, 1.045) | 0.967 (0.899, 1.041) |
|  | Proportion of elderly population (in %) |  | 0.866 (0.741, 1.012) | 0.924 (0.810, 1.053) |
|  | Distances to Wuhan (in 100 km) |  | 1.024 (0.957, 1.095) | 0.966 (0.913, 1.022) |
|  | Meteorological factors |  |  |  |
|  | Temperature (in ^o^C) |  | 0.901 (0.881, 0.921)** | 0.979 (0.956, 1.002) |
|  | Relative humidity (in %) |  | 0.991 (0.985, 0.996)* | 0.989 (0.984, 0.994)** |
|  | Control measure effect |  |  | 0.744 (0.724, 0.765)** |
|  | Time trend | 1.006 (1.002, 1.011)* | 1.007 (1.003, 1.012)* | 1.233 (1.206, 1.260)** |
|  | χ^2^/*df* | 0.36 | 0.34 | 0.13 |
|  | *R^2^_fixed_* | 0.48% | 14.8% | 51.3% |
|  | *R^2^_random_* | 27.8% | 27.0% | 11.3% |
|  | *∆R^2^_fixed_* | - | 14.4% | 50.8% |

Note: M1, Model with time only; M3, Model with city-specific characteristics, vapour pressure, and time M5, Model with city-specific characteristics, vapour pressure, control measure variable, and time (full model). RR, Relative risk in incidence rate of COVID-19 for each unit change of variable; χ^2^/*df*, chi-square statistics divided by the degree of freedom; *R^2^_fixed_*, Proportion of variance in the incidence rate (per million population) explained by the fixed effect terms; *R^2^_random_*, Proportion of variance explained by the random effect term of cities’ heterogeneity. *∆R^2^_fixed_*, *R^2^_fixed_* of each model minus *R^2^_fixed_* of M1.

**p*<0.05; ***p*<0.001

Details have been added to methods, results, and discussion:

“*A stratified analysis by climate zone was conducted to examine the difference in proportion of variance explained by factors between temperate and subtropical/tropical cities. Of the 102 included Chinese cities, 45 located in the temperate zone and 57 located in the subtropical or tropical zones.*” (Track change: Paragraph 4 in Statistical analysis)

“*While the analysis was stratified by climate zone, the additional variances explained by the control measures were similar between the temperate and subtropical/tropical cities when comparing with the variance explained in the model without the effects of M3 control measure (Table 4). However, the contribution of meteorological factors in the explained variance of the subtropical/tropical cities was around 3-fold more than that in the temperate cities (i.e. ∆R^2^_fixed_=14.4% vs ∆R^2^_fixed_=5.04% in M3). The temperature and relative humidity even became statistically insignificant in the full model when fitting the data of temperate cities (temperature: RR=0.981, 95% CI: 0.952-1.010, p=0.198; relative humidity: RR=0.997, 95% CI: 0.990-1.004, p=0.458).”* (Track change: Paragraph 6 in Results)

“*Instead, the implementation of control measures was associated with a larger proportion of variance explained with regard to the activity of COVID-19 and the result was robust to variations in climate zones of the cities and lag effects of meteorological factors.”* (Track change: Paragraph 1 in Discussion)

8. The study concludes that the impact of meteorology in the incidence of COVID 19 is limited. In addition to that, it is indicated in lines 227-228 that city-specific characteristics do not explain much of the variance in incidence rate. The control measures were launched more or less at the same time. What is then the main driver of the different incidence patterns observed in the studied cities and shown in Figure 2D? What is really driving the incidence? Has your model been available to detect that? The conclusion (last paragraph of the manuscript) indicates that “only the effect of control measures could explain a large portion of variability in COVID-19 activity”. Can we conclude then that the different patterns (phase and amplitude) observed in Figure 2D are basically explained by different levels of efficiency of the control measures (and the random aspect)?

**Response**: Thanks for your comment. Our study aims to demonstrate how much variability in COVID-19 activity was attributable to city-level socio-demographic characteristics, meteorological factors, and control measures of level I responses in order to confirm our study hypothesis that impact of meteorological factors was limited and only the control measures could control the COVID-19 epidemic. Apart from this objective, we did not explicitly identify which factor(s) was/were really driving the incidence since the variables we collected are not in sufficient detail. For example, we were unable to obtain the metrics related to healthcare capacity as well as the compliance/efficiency level of each control measure (i.e. recommended or mandatory). In addition, other methods such as boosted regression tree are more common in assessing the relative contributions among the factors (Chong et al. 2020; Gilbert et al. 2014.). Because of these reasons, we regret to say that the objective proposed is beyond our study focus.

Reference:

Chong KC, Lee TC, Bialasiewicz S, et al. Association between meteorological variations and activities of influenza A and B across different climate zones: a multi-region modelling analysis across the globe. J Infect. 2020;80(1):84-98.

Gilbert M, Golding N, Zhou H, Wint GW, Robinson TP, Tatem AJ, Lai S, Zhou S, Jiang H, Guo D, Huang Z. Predicting the risk of avian influenza A H7N9 infection in live-poultry markets across Asia. Nature communications. 2014 Jun 17;5(1):1-7.
